# A Meta-Analysis of Spurious Associations between Type D Personality and Cardiovascular Disease Endpoints

**DOI:** 10.1007/s12160-012-9356-7

**Published:** 2012-03-22

**Authors:** Jacob N. de Voogd, Robbert Sanderman, James C. Coyne

**Affiliations:** 1 Health Psychology Section, Department of Health Sciences, University Medical Center Groningen, University of Groningen, Groningen, the Netherlands; 2 Department of Pulmonary Rehabilitation, Center for Rehabilitation, University Medical Center Groningen, Groningen, the Netherlands; 3 Department of Psychiatry, University of Pennsylvania School of Medicine, 3535 Market St. Rm 676, Philadelphia, PA 19104 USA

Denollet et al. claim that high social inhibition and negative affectivity, termed type D personality, predict negative cardiovascular disease outcomes, and notably mortality [1]. Our group noted that almost all research testing this hypothesis came from small studies from Denollet’s group at Tilburg University that for mortality had 4, 6, 8, 12, and 47 deaths to explain. We conducted the largest study of mortality to date, with 192 deaths being explained, recruited patients from some of the same hospitals used by the Tilburg group, but found no association between type D personality and mortality [2]. Our primary analyses retained the component social inhibition and negative affectivity as continuous variables and tested their interaction. Secondary analyses relied upon Denollet’s preferred method of dichotomizing social inhibition and negative affectivity, creating a 2 × 2 cross-tabulation, and then comparing the high/high type D quadrant to the other three. Our results were still null but we drew on our own work [3] and other sources [4] to caution that Denollet’s strategy was inappropriate and likely to produce spurious results, as did an accompanying editorial [5].

We provided data from our study to Grande et al. for inclusion in their meta-analyses [6]. Consistent with what they had done in all but one of their past and forthcoming studies, Grande et al. relied on Denollet’s strategy of data reduction and analysis, disregarding our caution that type D personality data is frequently inappropriately analyzed in this manner is insufficient reason for continuing to do so.

In a further meta-analysis, Grande et al. combined multivariate analyses from Denollet et al. that consistently exceeded the number of allowable covariates, making spurious results likely. Problems in the credibility and generalizability of such overfit regression equations [7] are not overcome by simply integrating their results.

Grande et al. found substantial heterogeneity and evidence of publication bias in their meta-analysis and should have further pursued that this was due to what they termed a striking rift between “very large effects in small studies and low or null effects from the largest studies,” [6] which were conducted outside the Tilburg group. The rate of positive findings in the Tilburg studies is considerably beyond what would have been predicted by a power analysis based on events being explained. Figure 3 in the meta-analysis article is misleading and obscures this problem, but the pattern is readily apparent in an examination of the meta-analysis' other forest plots and tables.

Only after presenting their analyses, do Grande et al. concede “Our review cannot overcome some serious methodological shortcomings in Type D research,” Grande et al. then cataloged many of the criticisms of this research that we [2] had previously noted. Yet, they neither refuted these criticisms nor allowed them to influence how they conducted their meta-analysis. Instead, they ended with a declaration of the “urgency” for better studies of type D personality.

Our assessment is that this literature is indeed ailing, but we believe that pulling the plug is better than keeping it on life support. There simply is no encouragement from adequately powered studies conducted outside the Tilburg group to continue this line of research. This would be apparent in a meta-analysis that used an appropriate statistical approach in calculating type D and accurately pointed to the source of the publication bias in studies sharing some characteristics: positive results from small samples, inappropriate statistics, and having been conducted by a single investigator group.
